# P-447. Anal Pap Smear Trends in Patients Living with HIV and History of Malignancy in a Single-Center Retrospective Observational Study

**DOI:** 10.1093/ofid/ofae631.647

**Published:** 2025-01-29

**Authors:** Lauren E Rybolt, Ana Velez, Shivan Shah, Yanina Pasikhova, Shylah Moore Pardo, Anna Coghill, Jessica Islam, John Greene

**Affiliations:** University of South Florida, Tampa, FL; University of South Florida, Tampa, FL; University of South Florida, Tampa, FL; Moffitt Cancer Center, Tampa, Florida; University of South Florida, Tampa, FL; Moffitt Cancer Center & Research Institute, Tampa, Florida; Moffitt Cancer Center & Research Institute, Tampa, Florida; Moffitt Cancer Center, Tampa, Florida

## Abstract

**Background:**

The elevated rates of squamous cell carcinoma of the anus (SCCA) in persons living with HIV (PLWH) have led to greater awareness for screening with anal Pap cytology. While there is a premise that higher states of immunosuppression carry a greater risk of developing SCCA, no current studies describe this in the setting of PLWH with a history of solid or hematologic malignancy. The objective of this study is to review outcomes of anal Pap screenings in PLWH and a history of either solid or hematologic malignancies. With this study, we aim to outline potential risk factors associated with anal dysplasia in PLWH and a history of cancer.

**Figure:**
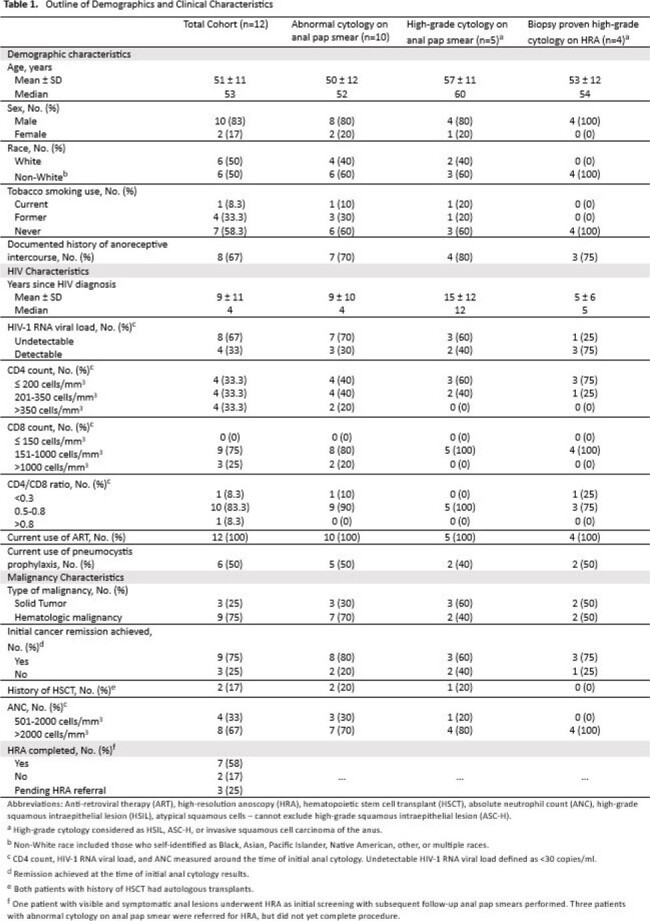

**Methods:**

This single-center retrospective cohort study included patients ≥ 18 years of age with a history of solid or hematologic malignancy who were followed at a cancer center clinic for HIV care between 2020-2024 and underwent anal Pap screening during this period. Demographics, pathology results, and clinical characteristics were obtained by chart review.

**Figure:**
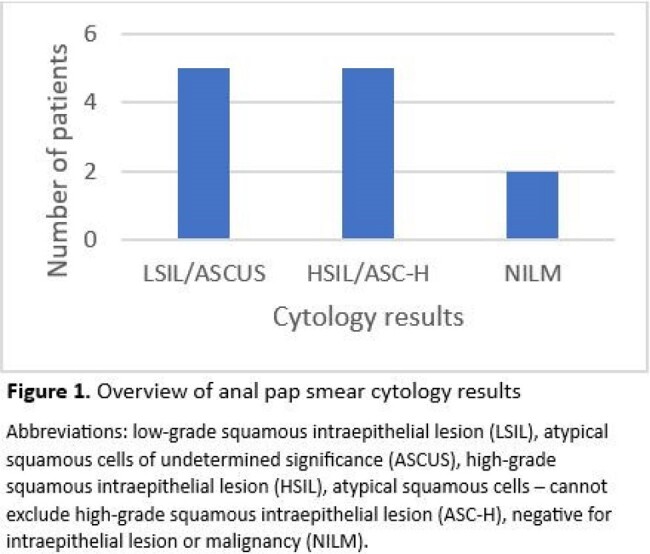

**Results:**

The study identified 12 patients to date, with a mean age of 51 and male-to-female ratio of 5:1. Abnormal cytology on anal Pap was present in 83.3% (n=10) of patients with 50% (n=5) of these abnormal screenings resulting in either high-grade squamous intraepithelial lesion (HSIL) or atypical squamous cells, cannot rule out high-grade squamous intraepithelial lesion (ASC-H). Most with abnormal cytology on anal Pap underwent high-resolution anoscopy (HRA) (n=7/10). High-grade cytology was present on biopsy in 57.1% of patients (n=4/7). Invasive SCCA was found during HRA in 1 patient who had ASC-H on anal Pap screening. Table 1 outlines demographic information and clinical characteristics. Figure 1 demonstrates cytology results.

**Conclusion:**

Over 75% screened had abnormal cytology, emphasizing the high incidence of anal dysplasia in this set of PLWH and a history of malignancy. The percentages of abnormal cytology in this population with a history of malignancy appeared to be higher than in other studies, implying chronic ongoing immune activation, although future work to expand the cohort is needed for robust estimation. These results should prompt further investigation to understand the potential interplay of HIV and malignancy history on the risk for developing anal dysplasia.

**Disclosures:**

**All Authors**: No reported disclosures

